# Counter Anion Type Influences the Glass Transition Temperature of Polyelectrolyte Complexes

**DOI:** 10.1021/acs.macromol.3c02200

**Published:** 2024-05-09

**Authors:** Suvesh Manoj Lalwani, Kayla Hellikson, Piotr Batys, Jodie L. Lutkenhaus

**Affiliations:** † Artie McFerrin Department of Chemical Engineering, Texas A&M University, College Station, Texas 77843, United States; ‡ Department of Materials Science and Engineering, Texas A&M University, College Station, Texas 77840, United States; § Jerzy Haber Institute of Catalysis and Surface Chemistry, Polish Academy of Sciences, Niezapominajek 8, Krakow PL-30239, Poland

## Abstract

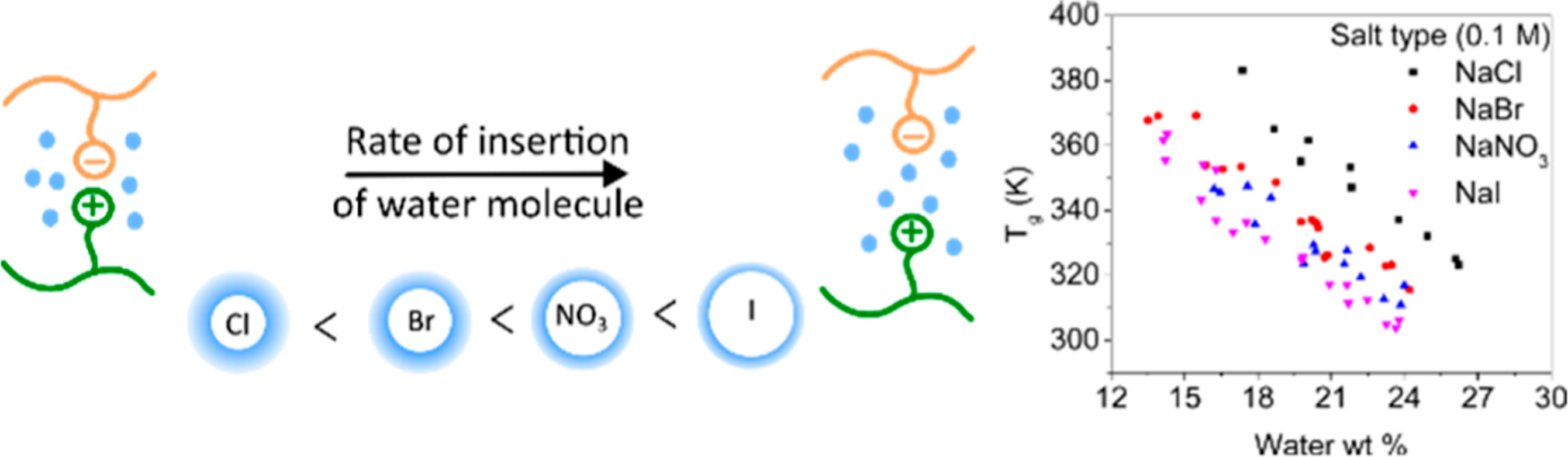

Salt acts as a plasticizer in polyelectrolyte complexes (PECs), which impacts the physical, thermal, and mechanical properties, thus having implications in applications, such as drug delivery, energy storage, and smart coatings. Added salt disrupts polycation–polyanion intrinsic ion pairs, lowering a hydrated PEC’s glass transition temperature (*T*_g_). However, the relative influence of counterion type on the PEC’s *T*_g_ is not well understood. Here, the effect of anion type (NaCl, NaBr, NaNO_3_, and NaI) on the *T*_g_ of solid-like, hydrated PECs composed of poly(diallydimethylammonium) (PDADMA)–poly(styrenesulfonate) (PSS) is investigated. With increasing the chaotropic nature of the salt anion, the *T*_g_ decreases. The relative differences are attributed to the doping level, the amount of bound water, the mobility of water molecules within the PECs, and the strength of interactions between the PEs. For all studied salt concentrations and salt types, the *T*_g_ followed the scaling of −1/*T*_g_ ≈ ln([IP]/[H_2_O]), in which [IP]/[H_2_O] is the ratio of intrinsic pairs to water. The scaling estimates that about 7 to 17% of the intrinsic ion pairs should be weakened for the PEC to partake in a glass transition. Put together, this study highlights that the *T*_g_ in PECs is impacted by the salt anion, but the mechanism of the glass transition remains unchanged.

## Introduction

Polyelectrolyte complexes (PECs) are formed as a result of phase separation when two solutions of oppositely charged polyelectrolytes (PEs) are mixed together.^1,2^ There are two phases formed: a polymer-rich phase containing the PEC and a dilute phase known as the supernatant. PECs can exist as solid-like complexes or liquid-like coacervates.^3−6^ The formation of PECs is driven by electrostatic attraction between oppositely charged PEs and entropic gain due to release of counterions and water molecules during complexation.^7^ The phase behavior and the thermal and mechanical properties are affected by water, temperature, pH, salt, solvent, hydrophobicity, and chemistry of the PEs.^8−27^ PECs are composed of two different types of ion pairs: extrinsic ion pairs formed between polyelectrolyte charge groups and counterions and intrinsic ion pairs formed between the oppositely charged PEs.^9^ Due to their tunability and versatility, PECs find applications in the field of drug delivery, underwater contact adhesives, separation membranes, electrochemistry, tissue engineering, and many more.^28−44^

Water acts as a plasticizer for solid PECs and promotes a transition from a glassy to a rubbery state.^2,45^ Huang et al. studied the mechanical properties of PEC fibers composed of alginate and poly(diallyldimethylammonium) (PDADMA) as a function of water content;^46^ they observed a glassy–rubbery transition with increasing water content. We studied the effect of water on the mechanical properties and relaxation times of poly(acrylic acid) (PAA)–poly(allylamine hydrochloride) (PAH) PECs,^12,13^ and we observed a relaxation time (τ) corresponding to the relaxation of multiple intrinsic ion pairs. τ was directly proportional to the ratio of intrinsic ion pairs (*n*_intrinsic ion pairs_) to water molecules (*n*_H_2_O_), . Thus, water present at the intrinsic ion pair helps in relaxation of PECs.

Water and salt together impact the glass transition temperature (*T*_g_) of PECs.^8,9,45,47^ We have studied the influence of water on the *T*_g_ of PAH–PAA^8^ and PDADMA–poly(styrenesulfonate) (PSS)^9^ PECs and polyelectrolyte multilayers (PEMs)^48,49^ with varying pH and ionic strength, and, in all cases, the *T*_g_ decreased with an increasing water content. Salt also acts as a plasticizer in PECs, converting an intrinsic ion pair to an extrinsic ion pair, which is termed as doping.^50^ For example, we found that the *T*_g_ of hydrated PDADMA–PSS PECs decreased with increased salt concentration due to a decrease in intrinsic ion pairing.^9^ Controlling the doping level has enabled PECs to be extruded as “saloplastics”.^51^ Taken together, the PEC’s *T*_g_ can be described by the ratio of water to intrinsic ion pairs^8,12^
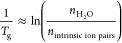
1

The studies that yielded eq 1 considered only the doping effects of KBr and NaCl. In one case, the *T*_g_ for PEMs assembled in KBr was less (20–25 °C) than that for PEMs assembled in NaCl at similar hydration levels.^48^ This difference was attributed to changes in the fraction of intrinsic ion pairs and differences in the internal structure of the PEMs. Overall, the effect of salt type on the *T*_g_ of a PEC is not well understood, especially because only a few assembly salts have been studied.

In our recent work,^52^ we explained the significance of eq 1 based on the insertion of a water molecule into the hydration shell of an intrinsic ion pair, which may lead to a brief separation or weakening of the intrinsic ion pair. This idea has parallels to the hypothesis^53^ that thermal transitions in PECs can result from changes in contact and solvent-separated ion pairs. The equilibrium constant between the intrinsic ion pair and the briefly separated intrinsic ion pair is given by *K*_eq_
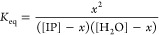
2where [IP] and [H_2_O] represent the concentration of intrinsic ion pairs and water molecules, respectively, and *x* represents the change in the concentration of intrinsic ion pair due to the insertion process. We speculated that “a *T*_g_-like event occurs when there exists a sufficient fraction of briefly separated intrinsic ion pairs, *f* (*f* = *x*/[IP])”.^52^ Combining this with eq 2 and the van’t Hoff equation yielded
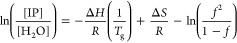
3where Δ*H* and Δ*S* represent the respective enthalpy and entropy changes. A comparison of eqs 1 and 3 shows that a plot between  versus  or  versus  should provide the enthalpy associated with the conversion of an intrinsic ion pair to a briefly separated intrinsic ion pair. Indeed, our previous work^8,9,48^ measured enthalpies of −10.5 to −11.5 kJ/mol, which were close to the enthalpy associated with the disruption of an H–O···H bond (10.5 ± 2.5 kJ/mol).^54^

In 1888, Hofmeister studied the solubility of egg-white proteins in different salt solutions and ranked the anions based on their tendency to precipitate protein.^55^ The series for monovalent anions goes as follows: OH^–^ < F^–^ < HCOO^–^ < CH_3_COO^–^ < Cl^–^ < Br^–^ < NO_3_^–^ < I^–^ < SCN^–^ < ClO_4_^–^. The ions to the left of chloride are kosmotropic (water-structuring or salting-out) and have a tendency to precipitate protein.^56^ These ions have a small size, well-ordered large hydration shell, and high electric field. On the contrary, the ions to the right of chloride are chaotropic (water-breaking or salting-in) and tend to solubilize protein. These ions have large size, loose, and small hydration shell and low electric field.

There have been various reports studying the effect of anion type on the thermodynamics of complexation, doping, and phase behavior of PECs.^7,50,57−59^ For example, Ghostine et al. studied the doping of PDADMA–PSS PECs for different anions along the Hofmeister series. Ion doping was found to increase with decreasing hydration of the anion.^50^ In a different study, Perry et al. studied the effect of anions on the phase behavior of PECS.^58^ The authors found that the critical salt concentration increased for kosmotropic ions (SO_4_^2–^, I^–^) when compared to Cl^–^. Taken together, anion effects on the phase behavior of PECs have been studied, but similar effects on the glass transition temperature in PECs have been largely overlooked.

Here, we examine the effect of anion type and hydration on the *T*_g_ of PDADMA–PSS PECs. The PECs are prepared with different salts: NaCl, NaBr, NaNO_3_, and NaI at different salt concentrations (0.1 M, 0.5 M). The composition of the individual PECs is determined using proton nuclear magnetic resonance (^1^H NMR) spectroscopy and neutron activation analysis (NAA). The glass transition temperature of hydrated PECs assembled in different salt types and concentrations is quantified using modulated differential scanning calorimetry (MDSC). Next, we identify the effect of anion type on the different states of water within the PECs using DSC and an analysis of the water’s freezing behavior. A connection between the *T*_g_ and the ratio of intrinsic ion pair concentration to the water concentration is made to evaluate the enthalpy associated with the *T*_g_. Finally, the effect of anion type on the *T*_g_ is discussed in terms of doping levels, water microenvironment, water diffusion, and PE–PE interaction strength.

## Experimental Section

### Materials

Poly(diallydimethylammonium) (PDADMA), 20 wt % in water, Mw = 200,000–350,000 g/mol was purchased from Sigma-Aldrich. Poly(sodium 4-styrenesulfonate) (PSS), *M*_w_ = 500,000 g/mol, was purchased from Scientific Polymer Products. Deuterium oxide and potassium bromide were purchased from Sigma-Aldrich. Sodium 2,2-dimethyl-2-silapentane-5-sulfonate (DSS), used as an internal NMR standard, was purchased from Cambridge Isotope Laboratories, Inc. Sodium chloride (NaCl, ACS reagent ≥99.0%), sodium bromide (NaBr, ACS reagent ≥99.0%), sodium iodide (NaI, ACS reagent ≥99.5%), and sodium nitrate (NaNO_3_, ACS reagent ≥99.0%) were purchased from Sigma-Aldrich. Milli-Q water with a resistivity of 18.2 MΩ·cm was used for all experiments.

### Preparation of PECs

Aqueous solutions of PDADMA and PSS at 50 mM concentration with respect to their repeat unit were prepared using Milli-Q water. The salt concentration in the PE solution was adjusted to either 0.1 M (NaCl, NaBr, NaNO_3_, and NaI) or 0.5 M (NaCl, NaBr, and NaNO_3_). The PECs were prepared by adding PSS into a solution of PDADMA during stirring. Due to the precipitation of PDADMA in 0.5 M NaI, it was not possible to prepare PECs at 0.5 M NaI. To evaluate the effect of the order of mixing, PECs were also prepared by adding PDADMA (0.1 M NaCl) into PSS (0.1 M NaCl). The resultant solution was stirred for 30 min at 600 rpm. The solid-like PECs were obtained after performing centrifugation at 10,000 rpm for 10 min. The precipitated PECs were then pressed at 6000 psi using a Carver Press and rinsed with copious water. The precipitates were then dried in a convection oven at 343 K and ground into a fine powder. The powdered PECs were then dried in a vacuum oven at 423 K for 6 h and sealed in a container until further use.

### Modulated Differential Scanning Calorimetry

MDSC (Q200, TA Instruments) was used to measure the *T*_g_ for the hydrated PECs. Hydrated samples were prepared by loading the dried PEC powder into a Tzero pan (TA Instruments) and by adding a known amount of solution corresponding to the preparation salt concentration and type to achieve a desired PEC hydration. The pan was subsequently sealed with a Tzero hermetic lid and was kept for at least 24 h before measuring the *T*_g_. MDSC was performed in a heat–cool–heat–cool cycle. The *T*_g_ was estimated from the second heating cycle unless otherwise stated. The samples were ramped from 278 to 393 K at a ramp rate of 2 K/min and an amplitude of 1.272 K for 60 s. Nitrogen was used as a purge gas at 50 mL/min. A typical sample loading (PEC + water) was 5–12 mg.

### Freezing Water Analysis

Differential scanning calorimetry (DSC) (Q200, TA Instruments), was used to identify different states of water within hydrated PECs as described previously.^60^ Hydrated samples for the measurement were prepared by loading dried PEC powder in a Tzero pan and adding the solution corresponding to the preparation salt concentration and type to achieve a PEC hydration of 24 wt %. DSC was performed in a cool–heat–cool–heat cycle. In brief, the hydrated samples were first cooled to 223 K and then heated to 293 at 5 K/min. Each ramp was followed by an isothermal step of 10 min. All DSC thermograms are shown in the “exotherm down” format and correspond to the second heating cycle unless otherwise stated.

The different states of water in PECs were classified according to the observed melting temperature of water, *T*_m_: (1) nonfreezing bound water, no *T*_m_; (2) freezing bound water, *T*_m_ < 273.15 K; and (3) freezing free water, *T*_m_ ≈ 273.15 K. The weight fraction of freezing water (*W*_f_) is the sum of freezing bound water (*W*_fb_) and the freezing free water (*W*_ff_)
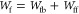
4
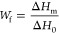
5where Δ*H*_m_ and Δ*H*_0_ represent the melting enthalpy of pure Milli-Q water and the observed melting enthalpy of water in the PECs, respectively. The melting enthalpy of pure Milli-Q water was 334 J/g.^60^ The melting enthalpy of water in the PECs was estimated from the integration of corresponding melting curve obtained from DSC. The weight fraction of the nonfreezing bound water, *W*_nf_, is given as
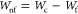
6where *W*_c_ is the hydration level of PECs.

### Neutron Activation Analysis

NAA is a highly sensitive technique used for elemental quantification. PEC powders weighing 20 to 30 mg were transferred into irradiation containers in a nitrogen glovebox to avoid moisture absorption. The samples were irradiated in a 1 MW TRIGA reactor for 30 s at a neutron flux of 9.1 × 10^12^ n/cm^2^ s and cooled for intervals of 270 s and up to 1 to 3 h. The released gamma ray photons were counted using a high-purity Ge (HPGe) gamma-ray detector. Elements Na, Cl, S, Br, and I were quantified using multiple gamma-ray emissions from the decay ^24^Na, ^38^Cl, ^37^S, ^80^Br, and ^123^I, respectively.

The wt % of S is an indication of the amount of PSS in the PECs. For PECs prepared with NaCl, NaBr, and NaI, the wt % of N is an indication of PDADMA. To calculate the amount of PDADMA, a mass balance was performed with the following assumptions: (1) the PEC was assumed to be electroneutral, PSS^–^ + Cl^–^ = PDAMA^+^ + Na^+^ and (2) the counterions were assumed to form extrinsic ion pairs. For PECs prepared with NaNO_3_, the wt % of N is an indication of the amount of NO_3_^–^ ions and PDADMA. In this case, the wt % of PDADMA was estimated using NMR spectroscopy, and the subsequent quantification of the NO_3_^–^ ions was done by performing a mass balance.

### Proton Nuclear Magnetic Resonance Spectroscopy

The mole ratio of PSS to PDADMA in the PECs was determined using proton nuclear magnetic resonance (^1^H NMR) spectroscopy (400 MHz proton frequency, Avance Neo 400 spectrometer). Dried complexes (50 mg) were dissolved in 1.5 mL 2.5 M KBr solution in D_2_O to fully dissolve the PECs to obtain a clear solution. The D_2_O peak at 4.79 ppm was chosen as the reference peak. The mole ratio of PSS to PDADMA was calculated by comparing the NMR peak areas corresponding to the 4 aromatic hydrogen of PSS (between 5.5 and 9.0 ppm), A_aro_, and the 3 aliphatic hydrogens of PSS and 16 aliphatic hydrogens of PDADMA (between 0.0 and 4.6 ppm), *A*_ali_.^16^ The mole ratio of PSS to PDADMA was determined using the following equation
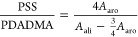
7

## Results

### PEC Composition and Doping Level

To understand the effect of anion type on the *T*_g_, it is important to first understand the molecular structure of the PEC, which is composed of intrinsic and extrinsic ion pairs. When salt is added to the PEC, intrinsic ion pairs are converted to extrinsic ion pairs, a process known as doping.^50^ The doping level is an indication of the fraction of extrinsic ion pairs within a PEC and depends on the salt concentration and salt type.

The composition of the PECs was determined using ^1^H NMR spectroscopy. PECs were prepared at a 1:1 PDADMA/PSS ratio by the repeat unit, centrifuged, and the solid complex precipitate was isolated. To calculate the composition, the isolated PECs were dissolved in 2.5 M KBr D_2_O solution, and the NMR spectra were recorded. Figure S1 shows the NMR spectra for PDADMA–PSS PECs prepared at different salt concentrations and different salt types. Due to the precipitation of PDADMA in 0.5 M NaI, it was not possible to prepare PECs at this desired salt concentration. As shown in Table S1, PDADMA was in excess at 53–57 mol % for all of the PECs. This suggests that the supernatant, by comparison, should be rich in PSS. For the case of PDADMA–PSS PECs prepared at 0.1 M salt concentration, the composition of the PECs varied slightly with the salt type. Specifically, PDADMA–PSS PECs prepared with NaCl, NaBr, and NaNO_3_ exhibited similar PDADMA concentrations of 53–54 mol %, but PECs prepared with NaI contained slightly more PDADMA at 57.6 mol %, as listed in Table S1. Elsewhere, we reported excess PDADMA for PECs prepared from NaCl solutions,^9^ which was ascribed to differences in the linear charge density and hydrophilicity between PSS and PDADMA, as well as the resulting counterion condensation.^61^

The doping level, which is related to the fraction of extrinsic ion pairs within the PEC, was estimated using elemental analysis via NAA and the preceding compositional analysis via NMR spectroscopy. Because of the asymmetric stoichiometry within the PECs, the doping levels for the polycation and polyanion will be dissimilar. The doping levels for PDADMA (y^+^) and for PSS (y^–^) are given by
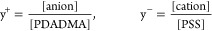
8where [anion], [cation], [PSS], and [PDADMA] represent the concentration of negative counterions, positive counterions, PSS repeat units, and PDADMA repeat units with the PEC, respectively. Because PSS is the limiting PE in the PEC, the concentration of intrinsic ion pairs, [IP], is given by
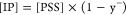
9

**Figure 1:**
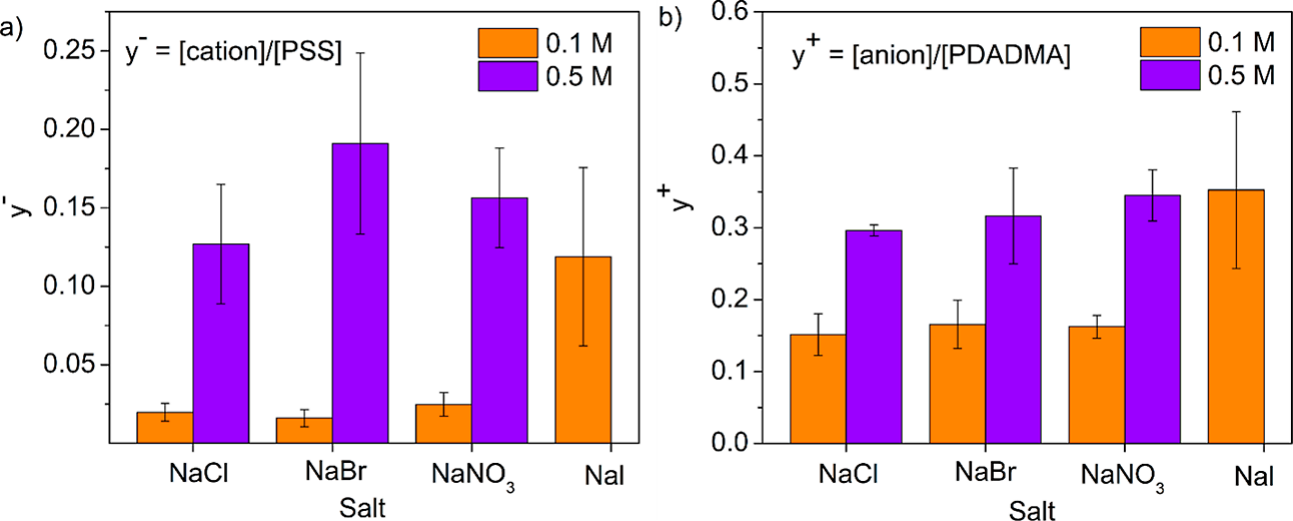
(a) PSS doping level (y^–^) and (b) PDADMA doping level (y^+^) measured via NAA for PDADMA–PSS PECs for different salt types.

Figure 1a,b shows the doping levels for PSS and PDADMA within the PECs, respectively. For a particular salt type, y^–^ and y^+^ increased with increasing salt concentration. As shown in Table S2, for a particular salt type, the counterion wt % increased, and the polymer (sulfur and nitrogen) wt % decreased with the increasing salt concentration, which is indicative of increased doping levels. For PECs prepared at 0.1 M salt concentration and different salt types, y^–^ and y^+^ were relatively constant for NaCl, NaBr, and NaNO_3_ at ∼0.02 and ∼0.15, respectively. Put another way, nearly every PSS unit and about 85% of PDADMA units participate in intrinsic ion pairing at 0.1 M. In comparison, y^–^ and y^+^ increased significantly to 0.1 and 0.35 for 0.1 M NaI, respectively; this indicates that 90% of PSS units and 65% of PDADMA units participate in intrinsic ion pairing at this condition. Overall, the doping levels for PECs prepared at 0.1 M salt concentration varied as Cl^–^ ≈ Br^–^ ≈ NO_3_^–^ < I^–^.

For PECs prepared at 0.5 M salt concentration, the doping levels were within error for the following anions: Cl^–^, Br^–^, and NO_3_^–^ but markedly higher than doping levels for PECs prepared at 0.1 M. Specifically, y^–^ and y^+^ were about ∼0.17 and ∼0.32, respectively, for PECs prepared at 0.5 M. This indicates that about 83% of PSS units and 68% of PDADMA units participate in intrinsic ion pairing at this condition. Taken together, increased salt concentration leads to increased doping levels with the PEC. Salt type, however, does not have a strong influence on the doping level, except for the case of NaI.

### Effect of Water and Ionic Strength on the *T*_g_ in PECs Prepared from NaBr

**Figure 2:**
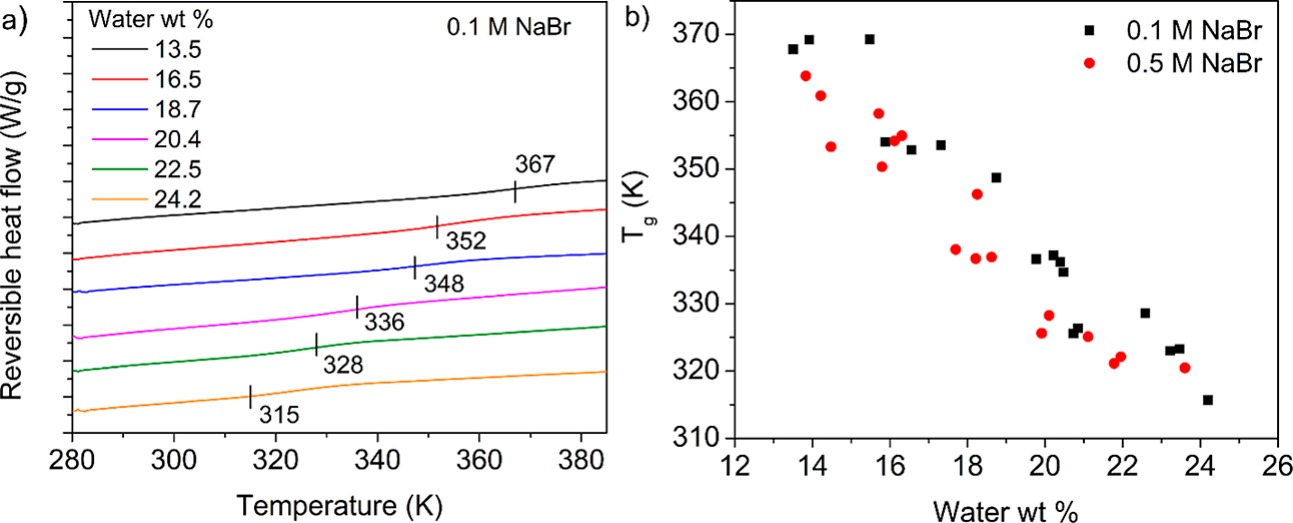
(a) Reversible heat flow curves (exotherm down) corresponding to the 2nd heating cycle at varying hydrations for PDADMA–PSS complexes prepared in 0.1 M NaBr. The curves are shifted vertically for clarity. (b) Glass transition temperatures for PDADMA–PSS complexes prepared in 0.1 and 0.5 M NaBr at varying hydration values. Each data point represents a single experiment.

The effect of water on the *T*_g_ for PECs was evaluated using MDSC. Figure 2a shows the second heating cycle corresponding to different hydration values for PDADMA–PSS complexes prepared at 0.1 M NaBr. The *T*_g_ was estimated as the inflection point corresponding to the reversible heat flow curve. The *T*_g_ decreased from 367 to 315 K with a corresponding increase in hydration from 13.5 to 24.2 wt %. To understand the effect of salt concentration on the *T*_g_, PECs were prepared in 0.1 and 0.5 M NaBr. Figure S2 shows the reversible heat flow curves for 0.5 M NaBr, and Figure 2b shows the corresponding comparison of *T*_g_’s at different hydration values. The glass transition is subtle but reproducible upon cooling and bears a signature enthalpic event in the nonreversing heat flow curve. For all cases studied, the *T*_g_ decreased with increasing hydration of the PEC. Further, the *T*_g_ values for PECs prepared from 0.5 M NaBr were lower than those prepared from 0.1 M NaBr.

### Effect of Anion Type on the *T*_g_

**Figure 3:**
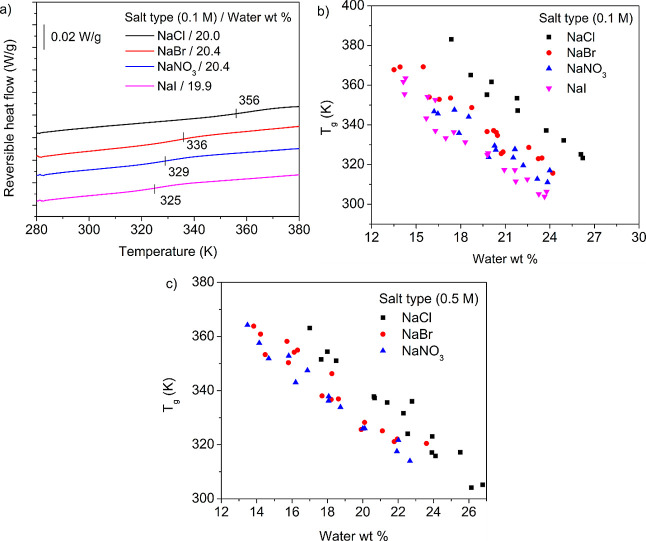
a) Reversible heat flow curves (exotherm down) corresponding to the 2nd heating cycle at ∼20 wt % hydration for PDADMA–PSS complexes prepared in 0.1 M NaCl, NaBr, NaNO_3_, and NaI. Glass transition temperatures for PDADMA–PSS complexes prepared with different salt types at (b) 0.1 M or (c) 0.5 M salt concentration and with varying hydration values. The curves on panel (a) are shifted vertically for clarity.

The effect of anion type on the *T*_g_’s of isolated PDADMA–PSS complexes prepared from NaCl, NaBr, NaNO_3_, and NaI were studied using MDSC for varying hydration values. Figure 3a shows the reversible heat flow for PECs hydrated at 20 wt % and prepared from 0.1 M salt concentration. PECs prepared in NaCl had the highest *T*_g_ of 356 K whereas PECs prepared in NaI had the lowest *T*_g_ of 325 K. Figure 3b shows the *T*_g_ as a function of hydration for different salt types at 0.1 M salt concentration. The *T*_g_ decreased with increasing hydration for all of the different salt types. Additionally, the *T*_g_ varied with the salt type as NaCl > NaBr > NaNO_3_ > NaI for a given hydration value. Interestingly, as seen in Figure 1a,b, the doping levels for PECs prepared in 0.1 M NaCl, NaBr, and NaNO_3_ were within error of each other. This indicates that, even if the doping levels are similar for NaCl, NaBr, and NaNO_3_, the type of anion can still affect the dynamics and *T*_g_ of the PECs. Figure 3c shows the variation in *T*_g_ for hydrated PECs prepared at the higher salt concentration of 0.5 M. It was observed that the *T*_g_ decreased as the salt concentration increased from 0.1 to 0.5 M, but the *T*_g_ followed the same trend with regard to the salt type, where *T*_g_ varied as NaCl > NaBr ≈ NaNO_3_. The general decrease in *T*_g_ for the higher salt concentration of 0.5 M is attributed to the increased doping levels, as shown in Figure 1a,b.

### Nonfreezing Bound, Freezing Bound, and Freezing Free Water in PECs

Water within a PEC can exist in different states caused by interactions with other polyelectrolytes and/or counterions, manifesting in an altered melting behavior.^60,62^ Specifically, water microenvironments may be classified as nonfreezing bound water, *W*_nf_ (no *T*_m_); freezing bound water, *W*_fb_ (*T*_m_ < 273.15 K); and freezing free water, *W*_ff_ (*T*_m_ ≈ 273.15 K).

**Figure 4:**
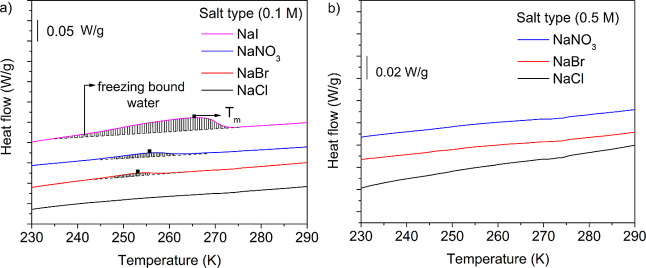
DSC heat flow curves (exotherm down) corresponding to the 2nd heating cycle of PECs prepared in different salts at (a) 0.1 and (b) 0.5 M and 24 wt % hydration. The curves are shifted vertically for clarity.

Here, we explored the different states of water at 24 wt % hydration for PDADMA–PSS PECs prepared in NaCl, NaBr, NaNO_3_, and NaI. At 0.1 M, no melting peak was observed for the PECs prepared in NaCl, suggesting that there was only nonfreezing bound water present, as shown in Figure 4a. In contrast, we observed a melting peak with *T*_m_ < 273.15 K for PDADMA–PSS PECs prepared in NaBr, NaNO_3_, and NaI, indicated that there was freezing bound water present. The *T*_m_’s for PECs prepared in NaBr and NaNO_3_ were similar (*T*_m_ = 251–256 K), whereas the *T*_m_ changed significantly for PECs prepared in NaI (*T*_m_ = 264 K), as listed in Table 1. From eqs 5 and 6, the weight percentage of freezing bound and nonfreezing bound water was calculated. For PECs prepared at 0.1 M, NaBr had the lowest wt % of freezing bound water (*W*_fb_ = 1.5 wt %) whereas NaI had the highest wt % of freezing bound water (*W*_fb_ = 8.0 wt %). In comparison, no freezing bound water was detected for PECs prepared in 0.5 M NaCl, NaBr, and NaNO_3_ at a similar hydration of 24 wt %, as shown in Figure 4b.

**Table 1:** Weight Percentages of Nonfreezing Water (*W*_nf_), Freezing Bound Water (*W*_fb_), Freezing Free Water (*W*_ff_), and Observed Melting Temperature (*T*_m_) of Water for PDADMA–PSS PECs at Different Salt Concentrations and Salt Types at 24 wt % Hydration

salt type/salt concentration (M)	*W*_nf_ (wt %)	*W*_fb_ (wt %)	*W*_ff_ (wt %)	*T*_m_ (K)
NaCl/0.1	23.5 ± 0.5	n/a^a^	n/a	n/a
NaCl/0.5	24.1 ± 0.2	n/a	n/a	n/a
NaBr/0.1	22.4 ± 0.5	1.5 ± 0.7	n/a	252.2 ± 1.0
NaBr/0.5	24.2 ± 0.3	n/a	n/a	n/a
NaNO_3_/0.1	20.4 ± 1.6	3.4 ± 1.6	n/a	254.4 ± 1.5
NaNO_3_/0.5	24.24 ± 0.01	n/a	n/a	n/a
NaI/0.1	8.0 ± 0.3	15.8 ± 0.3	n/a	264.7 ± 0.9

a n/a represents no detectable freezing bound water, freezing free water, or observed melting temperature.

### Relationship between *T*_g_ and the Ratio of Concentration of Intrinsic Ion Pairs to Water Molecules

**Figure 5:**
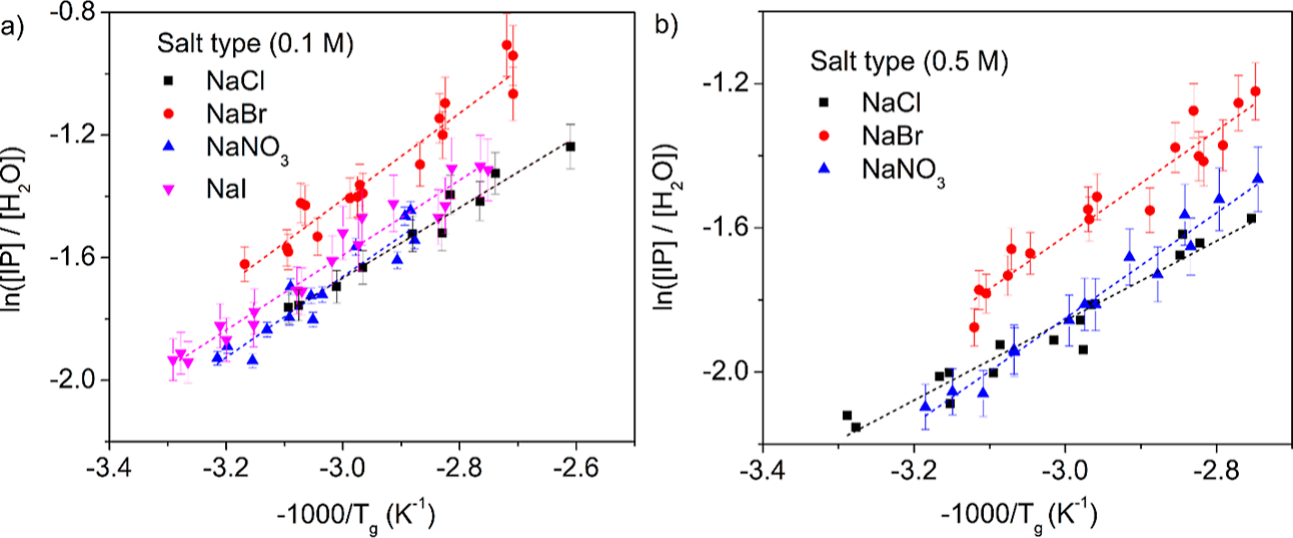
Plots of ln([IP]/[H_2_O]) vs −1000/*T*_g_ for PECs prepared in different salt types and salt concentrations of (a) 0.1 and (b) 0.5 M. The dotted lines show linear fits for each salt type, as listed in Table 2. Each data point represents a single experiment, and the error bars were calculated from the propagation of error associated with NAA.

In earlier reports from our group, the molar ratio of water molecules to intrinsic ion pairs was found to correlate to the *T*_g_ in PECs composed of strong and weak PEs, as shown in eq 1.^8,9,52^ Recently, we described the *T*_g_ based on an equivalent relationship between the *T*_g_ and the concentration ratio of intrinsic ion pairs and water ([IP]/[H_2_O]), as shown in eq 3.^52^ Accordingly, we replotted the *T*_g_’s from Figure 3 as ln([IP]/[H_2_O]) vs −1000/*T*_g_ for PECs prepared in 0.1 and 0.5 M salt solutions, as shown in Figure 5a,b, respectively. For all salt types investigated, linear trends were observed with *R*^2^ values of 0.89 to 0.97, as listed in Table 2. This relationship provided the van’t Hoff enthalpy associated with insertion of a water molecule into the intrinsic ion pair’s hydration shell. For all of the salts and concentrations examined, the enthalpy values were within error of each other, yielding an average enthalpy of 10.9 ± 0.5 kJ/mol. Remarkably, this is close to the enthalpy associated with a single hydrogen bond breaking (10.5 ± 2.5 kJ/mol).^54^

**Table 2:** Enthalpies and y-Intercepts for PECs Estimated from Linear Fitting of ln([IP]/[H_2_O]) vs −1000/*T*_g_. The Error Bars are Based on a 95% Confidence Interval of the Linear Fit

PECs	*R*^2^	enthalpy (kJ/mol)	*y*-intercept
0.1 M NaCl	0.97	9.6 ± 1.3	–1.8 ± 0.5
0.5 NaCl	0.94	9.2 ± 1.3	–1.5 ± 0.7
0.1 M NaBr	0.94	12.0 ± 1.5	–3.0 ± 0.6
0.5 NaBr	0.93	12.1 ± 1.8	–2.0 ± 0.7
0.1 M NaNO_3_	0.89	11.0 ± 2.4	–2.3 ± 0.9
0.5 M NaNO_3_	0.97	12.3 ± 1.2	–2.6 ± 0.5
0.1 M NaI	0.96	10.1 ± 1.0	–2.0 ± 0.4

The enthalpy (slope) and y-intercepts were within error for PDADMA–PSS PECs across 0.1 and 0.5 M for the same salt type, as listed in Table 2. Previously, we observed a collapse of *T*_g_ values when plotted in a similar way for PDADMA–PSS PECs across different NaCl concentrations.^9^ In comparison to our previous work, the collapse of our own data was not as perfect across different NaCl, NaBr, and NaNO_3_ at different salt concentrations, as shown in Figure S4. This may be due to errors associated with the calculation of intrinsic ion pairs, which primarily comes from error associated with measurement of the elemental composition of PECs from NAA. Also, this error may be due to the limited range of salt concentrations explored (here, 0.1 and 0.5 M vs a much broader range of 0, 0.1, 0.5, 1.0, and 1.5 M in ref (9)). Last another source of error could be assigned to the presence of co-ions. Yang et al. reported that salt ions entering the PEC can participate in extrinsic ion pairing with a PE or remain unassociated (i.e., as co-ions);^63^ they found that the fraction of salt ions acting as co-ions varied with the salt type. In this study, we do not estimate the fraction of co-ions and calculation of concentration of intrinsic ion pairs neglected the possibility of co-ions, as shown in eq 9. Thus, the y-intercepts maybe be different across different salt types and concentrations due to inaccuracy associated with the calculation of concentration of number of intrinsic ion pairs. However, we emphasize that the enthalpies and y-intercepts are, overall, within error of each other, so we caution a strong interpretation of differences among the values.

## Discussion

### Doping Levels

PECs have two different types of ion pairs, namely: intrinsic and extrinsic ion pairs. When salt is added to PECs, intrinsic ion pairs are converted to extrinsic ion pairs accordingly
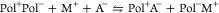
10

The doping level in PECs, eq 8, depends on the salt concentration and salt type. Figure 1a,b shows that the doping level increased with the salt concentration. This is due to conversion of intrinsic ion pairs to extrinsic ion pairs, eq 10. We and others also observed an increase in the doping level with increasing salt concentration for PDADMA–PSS PECs prepared in NaCl.^3,9^

As seen in Figure 1a,b, the doping level at 0.1 M preparation salt concentration varied as Cl^–^ ≈ Br^–^ ≈ NO_3_^–^ < I^–^ Additionally, the doping level at 0.5 M preparation salt concentration varied as Cl^–^ ≈ Br^–^ ≈ NO_3_^–^. Our results regarding doping levels across different salt types are similar to those reported by Ghostine et al. for PDADMA–PSS PECs.^50^ The authors measured the equilibrium doping level by immersing PECs in water and evaluating the water’s conductivity as a function of time. The authors reported a similar trend for doping: Cl^–^ ≤ NO_3_^–^ ≤ Br^–^ < I^–^. The differences in doping levels were attributed to variations in counterion hydration. Elsewhere, Rmaile et al. studied the doping level for PDADMA–PSS PEMs for different anions along the Hofmeister series.^64^ The authors reported that more hydrophobic anions yielded higher doping levels. Table 3 lists the hydration number for various anions used in this study. I^–^ has the lowest hydration number (highest hydrophobicity) and thus is the most efficient dopant.

**Table 3:** Compilation of Relevant Properties of Anions, Extracted from ref (65)

anion	ionic radius (nm)	hydrated radius (nm)	charge/area (e–/Å^2^)	hydration number	Jones–Dole coefficient	polarizability (Å^3^)
Cl^–^	0.181	0.319	0.0243	2.0	–0.005	8.57
Br^–^	0.196	0.337	0.0207	1.8	–0.033	12.22
NO_3_^–^	0.179	0.316/0.265/0.345 (mean/axial/equatorial)	0.0199	2.0	–0.045	10.59
I^–^	0.220	0.365	0.0171	1.6	–0.073	18.68

Taken together, the doping level increased with increase in salt concentration due to an increase in extrinsic ion pairing. Second, the doping level was the highest for PECs prepared in NaI due to I^–^ having the highest hydrophobicity.

### Influence of Water and Salt Concentration on the *T*_g_

Figure 2a,b shows that the *T*_g_ decreases with increasing hydration. Previously, we also reported a decrease in *T*_g_ for PECs composed of weak and strong PEs with added water.^8,9^ Water increases the free volume and facilitates the lubrication of the intrinsic ion pair resulting in lower *T*_g_ values.^13^ Additionally, water also decreases the strength of electrostatic attraction between the oppositely charged PEs, thus lowering an activation energy barrier for insertion of a water molecule into the hydration shell of intrinsic ion pair.^62,66^Figure 2b also shows that the *T*_g_ decreases with increasing salt concentration. An increase in the salt concentration leads to higher doping levels and a lower *T*_g_ due to the conversion of intrinsic ion pairs to extrinsic ion pairs. Previously, we also observed decreased *T*_g_ values with increased preparation salt concentrations for PDADMA–PSS PECs.^9^ To sum up, *T*_g_ decreased with increasing hydration due to the increased lubrication of PE chains, and *T*_g_ decreased with increasing preparation salt concentration due to the increased fraction of extrinsic ion pairs.

### Influence of Salt Type on the *T*_g_

Figure 3b shows that the *T*_g_ is dependent on the complex’s salt type, and the complexes’ *T*_g_ varied as follows at 0.1 M: NaCl > NaBr > NaNO_3_ > NaI for a given hydration value. However, it is noteworthy to recall that the PECs prepared from 0.1 M NaCl, NaBr, and NaNO_3_ had similar doping levels. Thus, differences in *T*_g_ do not arise solely due to differences in doping levels.

As previously shown, the behavior of water in a PEC influences the PEC’s macroscopic properties.^60^ We speculate that the water microenvironment may be altered due to the anion type, thus influencing the observed *T*_g_. Figure 4a and Table 2 describe the water microenvironment indirectly through the wt % of nonfreezing bound water (*W*_nf_) in the PEC. The wt % of nonfreezing bound water in the PECs varied as follows: NaCl > NaBr > NaNO_3_ > NaI. Recently, Gregory et al. reported that the Coulombic interaction between the anion and the corresponding solvent molecule is directly proportional to radial charge density, with the radial charge density decreasing as follows: Cl^–^ > NO_3_^–^ > Br^–^ > I^–^.^67^ Based on this, the columbic interaction strength between the anion and water molecule should be the highest in the case of PECs prepared in NaCl and lowest in the case of PECs prepared in NaI. This is in close agreement with the wt % of freezing bound water (*W*_fb_) which varied as NaI > NaNO_3_ > NaBr > NaCl. To compare, freezing bound water is expected to have relatively more mobility and degrees of freedom than nonfreezing bound water. We postulate that the freezing bound water may provide increased lubrication or lower energy barriers for the relaxation of PECs and, thus, lower *T*_g_ values.

The diffusion coefficient of water in the PEC may also play a role in the PEC’s relaxation. At the studied hydration levels, no free water was observed, suggesting that all water molecules are located in the first or second hydration shell of PE or ions. Therefore, water in the PEC is immobilized through the percolated hydrogen bond network. It has been shown recently^68^ that ions can affect the dynamics of water molecules beyond the first solvation layer by modifying the intermolecular interactions of water. Additionally, this effect depends on the ions’ polarizability. Ions with higher polarizability, such as I^–^, disrupt the hydrogen bond network to a greater extent when compared against ions with lower polarizability such as Cl^–^. This results in more mobile water molecules in the case of I^–^ as compared against Cl^–^. In a different study, Borkowski et al. studied the diffusion of water in 1 M sodium halide (NaCl, NaBr, and NaI) aqueous solutions using molecular dynamics;^69^ they found that the diffusion coefficient increased with the increasing chaotropic nature of the anion. If this ranking holds true in the environment of a PEC, the diffusion coefficient of water in PECs should vary as NaI > NaNO_3_ > NaBr > NaCl. Specifically, MD simulations of varying anions in PECs have not yet been conducted to verify this trend, but prior work may be considered. Batys et al. studied the microenvironments of water in different PECs and found that PECs with a higher percentage of freezing water are expected to exhibit higher water diffusion coefficients.^62^ Given our observations in Table 2, it is possible that the water diffusion may also vary according to *W*_fb_.

As mentioned before, we proposed that the *T*_g_ in PECs was driven by the insertion of water into the intrinsic ion pair’s hydration environment.^66^ If we consider this insertion as the rate determining step, a lower *T*_g_ should be observed for PECs exhibiting higher water diffusion because there will be more opportunities for water molecules to interact with the intrinsic ion pair. For example, PECs prepared in NaCl have only nonfreezing bound water and that water is expected to have a low diffusivity; as a result, PECs prepared in NaCl have the highest *T*_g_ of the salts studied. In contrast, PECs prepared in NaI exhibit the highest percentage of freezing bound water and that water is expected to have a higher diffusivity; as a result, PECs prepared from NaI have the lowest *T*_g_ of the salts studied.

Additionally, the strength of interactions between the oppositely charged PEs may also impact the *T*_g_. Yamazaki et al. studied the interaction strength between positively charged chondroitin sulfate C (CS), sodium salt, and negatively charged chitosan (CHI) in the presence of different monovalent salts (NaCl, NaBr, NaNO_3_, NaI, and NaSCN) using MD simulations.^70^ The authors found that the distance between the two charged polyelectrolytes was greater in the presence of chaotropic anions (I^–^ and SCN^–^) when compared to kosmotropic anions (Cl^–^). This finding^70^ indicates that chaotropic anions weaken intrinsic ion pairs more effectively. In our own work, the weakening of the PDADMA–PSS intrinsic ion pair is caused by chaotropic iodide, thus lowering the *T*_g_ relative to the more kosmotropic anions. Elsewhere, the strength of interaction between the two PEs in a PEC is also reflected by the critical salt concentration (CSC), which is the salt concentration at which there is no phase separation observed when the PEs are mixed. Perry et al. observed a decrease in CSC for PAH–PAA PECs in various salts, for which CSC decreased as NaCl > NaBr > NaI.^58^ In a different study, Spruijt et al. studied the CSC for poly(*N*,*N*-dimethylaminoethyl methacrylate)/PAA PECs in the presence of different monovalent salts.^71^ The CSC ranked as KCl > KNO_3_ > KBr > KI > KSCN. Thus, we conclude that larger anions with higher polarizability are more effective in disrupting intrinsic ion pairs. Similarly, we observed a lower *T*_g_ for PECs prepared with NaNO_3_ when compared to NaCl and NaBr even though the doping levels were similar. Taken together, when comparing PECs across different salt types with similar doping levels (i.e., 0.1 M NaCl, 0.1 M NaBr, and 0.1 M NaNO_3_), the difference in *T*_g_ can occur due to varying (a) wt % of freezing bound water, (b) diffusion of water molecules, and/or (c) intrinsic ion pair interaction strength.

### Van’t Hoff Enthalpy and Critical Fraction “F” Estimated from Relationship *T*_g_ and [IP]/[H_2_O]

As shown in Figure 5a,b, the *T*_g_, [IP], and [H_2_O] followed the linear relationship described in eq 3 for all different salt types investigated. According to Table 2, the van’t Hoff enthalpy estimated from the slope of eq 3 was within error for all salt types and salt concentrations of PEC preparation. The average enthalpy was 10.9 ± 0.5 kJ/mol, which was close to the enthalpy associated with a single water hydrogen bond (10.5 ± 2.5 kJ/mol).^54^ This highlights the role played by water in plasticizing the PECs and further supports the theory proposed by us that *T*_g_ in PECs is driven by insertion of water molecules.^52^

According to Table 2, the *y*-intercepts were found to be different across the different salt types, especially for PECs prepared in 0.1 M NaBr and 0.5 M NaBr. We find no particular trend for y-intercepts regarding the anion’s polarizability or hydration number. Additionally, the *y*-intercepts from eq 3 are given by
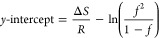
11

We further estimated the value of *f* (i.e., the fraction of briefly separated intrinsic ion pairs) using the entropy associated with the water hydrogen bond, Δ*S*/*R* = −2.4.^72^ The value of f varied from 0.07 to 0.17, but no particular trend was observed, as listed in Table S4. In general, this suggest that about 7 to 17% of the intrinsic ion pairs need to be weakened to undergo an observable glass transition relaxation.

As mentioned earlier, co-ions can skew the calculation of intrinsic ion pair concentration, thus leading to error in the y-intercept value for eq 3. As a consequence, adjustments in the *y*-intercept would influence the estimation of *f* (i.e., the fraction of briefly separated intrinsic ion pairs). Therefore, estimations of the co-ion concentration are needed, especially at higher concentrations, where the co-ion concentration is not insignificant. For example, co-ions were detected in PDADMA–PSS PECs at salt concentrations of 0.3–0.4 M.^63^

## Conclusions

In this work, we studied the effect of anion type and salt concentration on the doping level and *T*_g_ of PDADMA–PSS PECs. The *T*_g_ in PECs was impacted by the hydration level, salt concentration, and the identity of salt anions. As expected, the *T*_g_ decreased with increasing the PEC’s hydration level and with increasing the salt concentration for PEC assembly. Interestingly, anion type influenced the *T*_g_ in a Hoffmeister-type ranking. In the case of PECs prepared at 0.1 M, the *T*_g_ varied as follows: NaCl > NaBr > NaNO_3_ > NaI. To ascribe the reasons for this trend, we considered possible influences from differences in the (a) doping level, (b) wt % of freezing bound water, (c) diffusion of water molecules, and/or (d) strength of interaction between the PEs. Because the doping level did not vary strongly (except for NaI) for a given assembly concentration, we conclude that the doping level alone does not influence the *T*_g_. However, our studies on water’s microenvironment revealed that the wt % of freezing bound water followed the inverse ranking as the *T*_g_ for PECs was prepared in various salts at a concentration of 0.1 M. Specifically, PECs with more freezing bound water exhibited lower *T*_g_ values. This indicates that water’s microenvironment, in addition to the PEC’s doping level, influences the PEC’s *T*_g_. The scaling of −1/*T*_g_ ≈ ln([IP]/[H_2_O]) was found to be applicable across all of the different salt concentrations and salt types. The average enthalpy calculated from the scaling was close to the enthalpy associated with a hydrogen bond. To place these findings in the context of our previous work, the *T*_g_ in PECs is mediated by the diffusion of water molecules and their interaction with the intrinsic ion pair, which are impacted by the identity of the salt anion. Taken together, the results here show that the *T*_g_ in PECs is impacted by the salt anion, but, importantly, the mechanism of the glass transition relaxation in PECs remains unchanged. It remains unclear if this is true in the case of PECs prepared with different monovalent cations, divalent cations, and divalent anions. Additionally, the effect of different solvents on the *T*_g_ remains unexplored. This should provide more insights into how hydrogen bonding, dielectric environment, and other noncovalent interactions impact the *T*_g_ in PECs.

## Supporting Information Available

Mole % of PDADMA in PECs; ^1^H NMR spectra; weight % of different elements is PECs; modulated heating curve for PECs; ln([IP]/[H_2_O]) vs −1000/*T*_g_ for PECs; and critical fraction of intrinsic ion pairs for PECs (PDF)

## Abbreviations

PAA: poly(acrylic acid)
PAH: poly(allylamine hydrochloride)
PDADMA: poly(diallydimethylammonium)
PSS: poly(styrenesulfonate)
D_2_O: deuterium oxide
KBr: potassium bromide
NaCl: sodium chloride
NaBr: sodium bromide
NaNO_3_: sodium nitrate
NaI: sodium iodide
PE: polyelectrolyte
PECs: polyelectrolyte complexes
DSC: differential scanning calorimetry
MDSC: modulated differential scanning calorimetry
NAA: neutron activation analysis
MD: molecular dynamics
RH: relative humidity
CSC: critical salt concentration

## Nomenclature

τ: relaxation time of intrinsic ion pairs in hydrated PECs
ϕ_W_: volume fraction of water
*T*_g_: glass transition temperature
*N*_H_2_O_: number of water molecules
*n*_intrinsic ion pairs_: number of intrinsic ion pairs
*K*_eq_: equilibrium constant
[IP]: concentration of intrinsic ion pairs
[H_2_O]: concentration of water molecules
*x*: change in concentration of intrinsic ion pair
Δ*H*: enthalpy change associated with insertion of water molecule
Δ*S*: entropy change associated with insertion of water molecule
*R*: universal gas constant
*f*: fraction of briefly separated intrinsic ion pairs
*T*_m_: observed melting temperature of water
Δ*H*_m_: observed enthalpy of melting of frozen water
Δ*H*_0_: enthalpy of melting of pure Mili-Q water
*W*_f_: weight fraction of freezing water
*W*_c_: hydration level (%) of PECs
*W*_fb_: weight % of freezing bound water
*W*_ff_: weight % freezing free water
*W*_nf_: weight % nonfreezing bound water
*A*_aro_: area of aromatic hydrogens
*A*_ali_: area of aliphatic hydrogens
Y^+^: doping level for PDADMA
y^–^: doping level for PSS
[anion]: concentration of negative counterions in PECs
[cation]: concentration of positive counterions in PECs
[PSS]: concentration of PSS
[PDADMA]: concentration of PDADMA
*R*^2^: coefficient of determination
