# Supplementary material for: Counter Anion Type Influences the Glass Transition Temperature of Polyelectrolyte Complexes
Source: Macromolecules. 2024 May 9;57(10):4695–705. doi: 10.1021/acs.macromol.3c02200 (PMC11140738; doi:10.1021/acs.macromol.3c02200)
Supplement: Supplementary file 1 — ma3c02200_si_001.pdf [file ma3c02200_si_001.pdf]

Counter Anion Type Influences the Glass Transition Temperature of Polyelectrolyte Complexes

Suvesh Manoj Lalwani,[1] *Kayla Hellikson,*[1] *Piotr Batys,*[3] *Jodie L. Lutkenhaus*[1,2,*]

1. Artie McFerrin Department of Chemical Engineering, Texas A&M University, College Station, Texas 77843, United States

2. Department of Materials Science and Engineering, Texas A&M University, College Station, Texas 77840, USA

3. Jerzy Haber Institute of Catalysis and Surface Chemistry, Polish Academy of Sciences, Niezapominajek 8, PL-30239 Krakow, Poland

KEYWORDS: Polyelectrolyte, polyelectrolyte complexes, anion, Hofmeister, glass transition temperature, water

Corresponding Author

*Email address: jodie.lutkenhaus@tamu.edu

Table S1. Mole % of PDADMA estimated from area of aliphatic and aromatic hydrogens observed in ^{1}H-NMR spectroscopy.

PECs (Salt type/salt concentration (M))	PDADMA mole %
NaCl / 0.1	53.59±0.32
NaCl / 0.5	55.30±0.34
NaBr / 0.1	54.11±0.02
NaBr / 0.5	54.19±0.30
$NaNO_3$ / 0.1	53.78±0.05
$NaNO_3$ / 0.5	56.30±0.02
NaI / 0.1	57.64±0.24

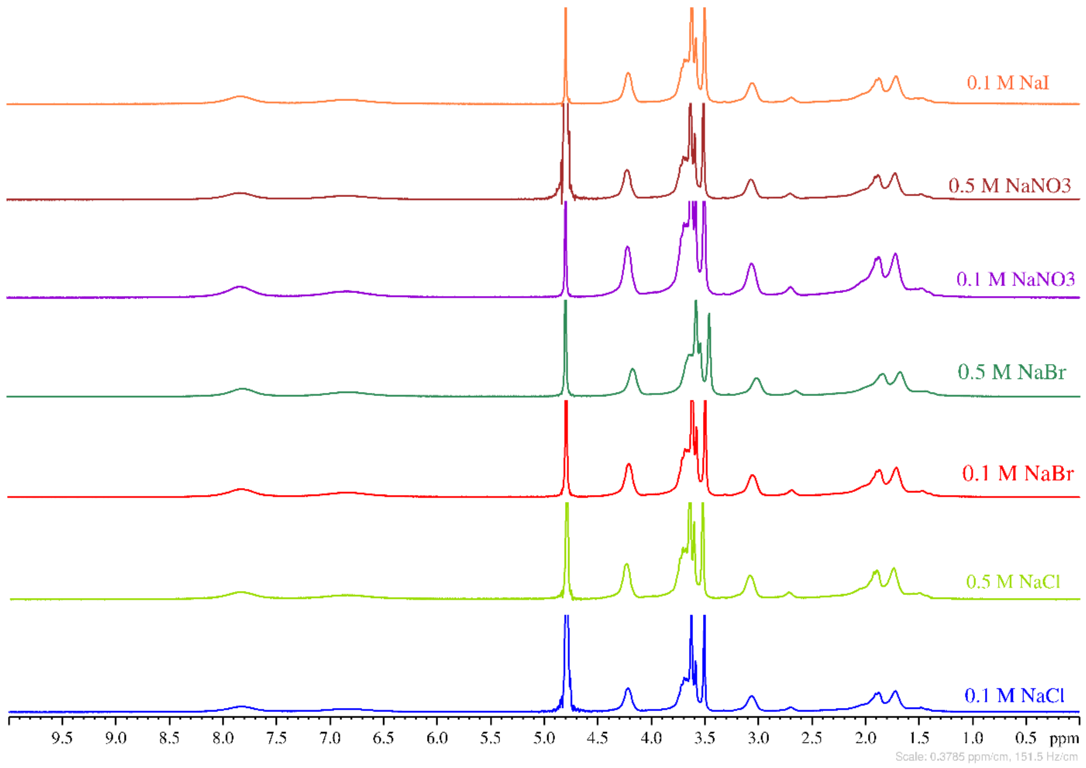

Figure S1. ^{1}H-NMR spectra of PECs in 2.5 M KBr in D_2O.

Table S2. Weight % of different elements in polyelectrolyte complexes (PECs) obtained from neutron activation analysis (NAA).

Salt type/Salt concentration (M)	Na (wt %)	Cl (wt %)	Br (wt %)	NO_3 (wt %)	I (wt %)	S (wt %)	N (wt %)
NaCl / 0.1	0.15±0.02	2.11±0.05	0	0	0	10.90±2.85	5.50±1.25
NaCl / 0.5	0.79±0.02	3.49±0.09	0	0	0	8.65±0.02	4.68±0.04
NaBr / 0.1	0.13±0.03	0.30±0.04	4.89±0.03	0	0	11.40±2.97	5.88±1.30
NaBr/ 0.5	1.43±0.09	0.16±0.03	9.36±0.05	0	0	10.40±3.07	5.38±1.34
$NaNO_3$ / 0.1	0.15±0.04	0.32±0.05	0	2.48	0	8.30±1.39	4.22±0.60
$NaNO_3$ / 0.5	0.85±0.06	0.12±0.02	0	6.32	0	7.60±1.44	4.28±0.63
NaI / 0.1	0.77±0.09	0.18±0.04	0	0	16.55±0.07	9.0±4.20	5.38±1.83

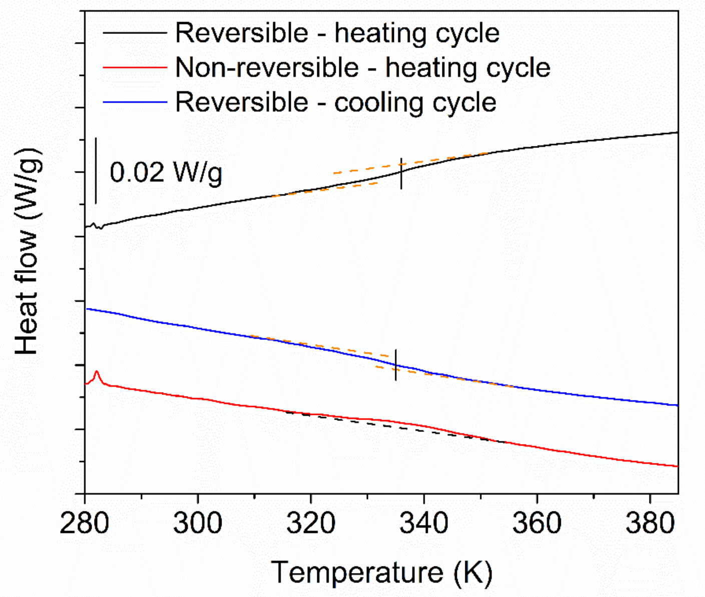

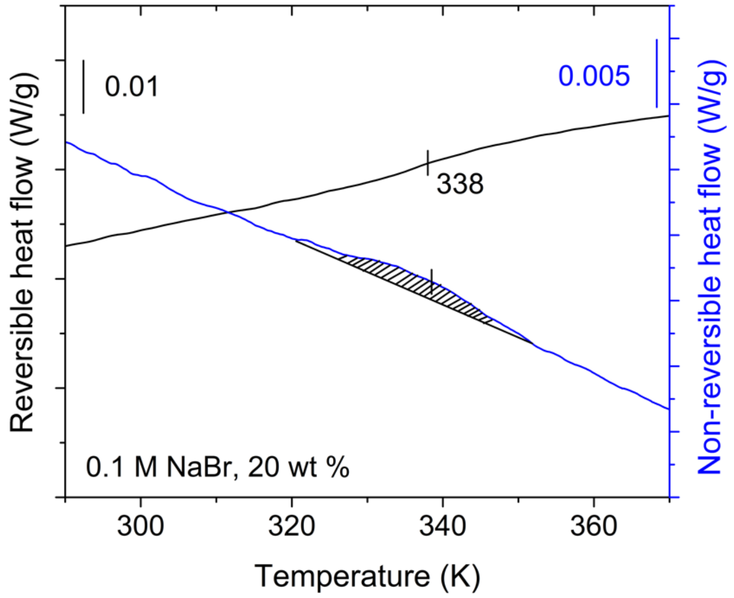

Figure S2. (Top) Modulated heating curve for PSS-PDADMA PECs prepared at 0.1 M NaBr at 20 wt % hydration. The black curve corresponds to the reversible heat flow for 2nd heating cycle. The red curve corresponds to the non-reversible heat flow for 2nd heating cycle. The inflection point for reversible heat flow corresponds to the peak observed in non-reversible heat flow. The blue curve represents the 2nd cooling curve. The inflection point is also evident in the cooling cycle. (Bottom) The T_g can be challenging to determine because it is so broad and weak (the change in C_p is slight). In addition to examining the inflection point in the reversing curve, it is helpful to inspect the non-reversing heat flow, which contains the enthalpic component of the T_g (*i.e.* physical aging response) as a broad peak occurring over the broad range of T_g.

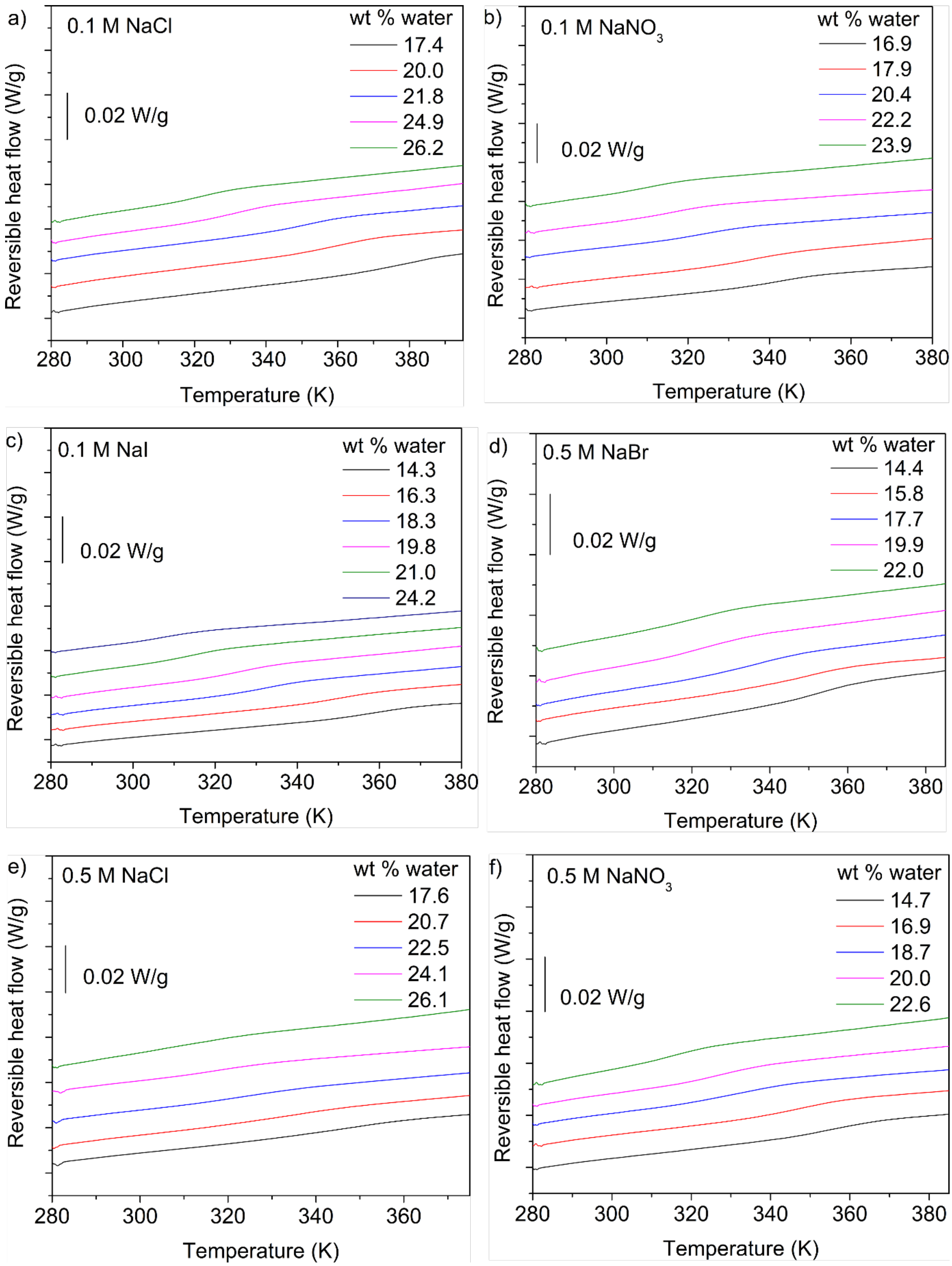

Figure S3. Reversible heat flow curves (exotherm down) corresponding to the 2nd heating cycle at varying hydrations for PDADMA-PSS complexes prepared in a) 0.1 M NaCl b) 0.1 M $NaNO_3$ c) 0.1 M NaI d) 0.5 M NaBr e) 0.5 M NaCl f) 0.5 M $NaNO_3$. The curves in all the above panels are shifted for vertical clarity.

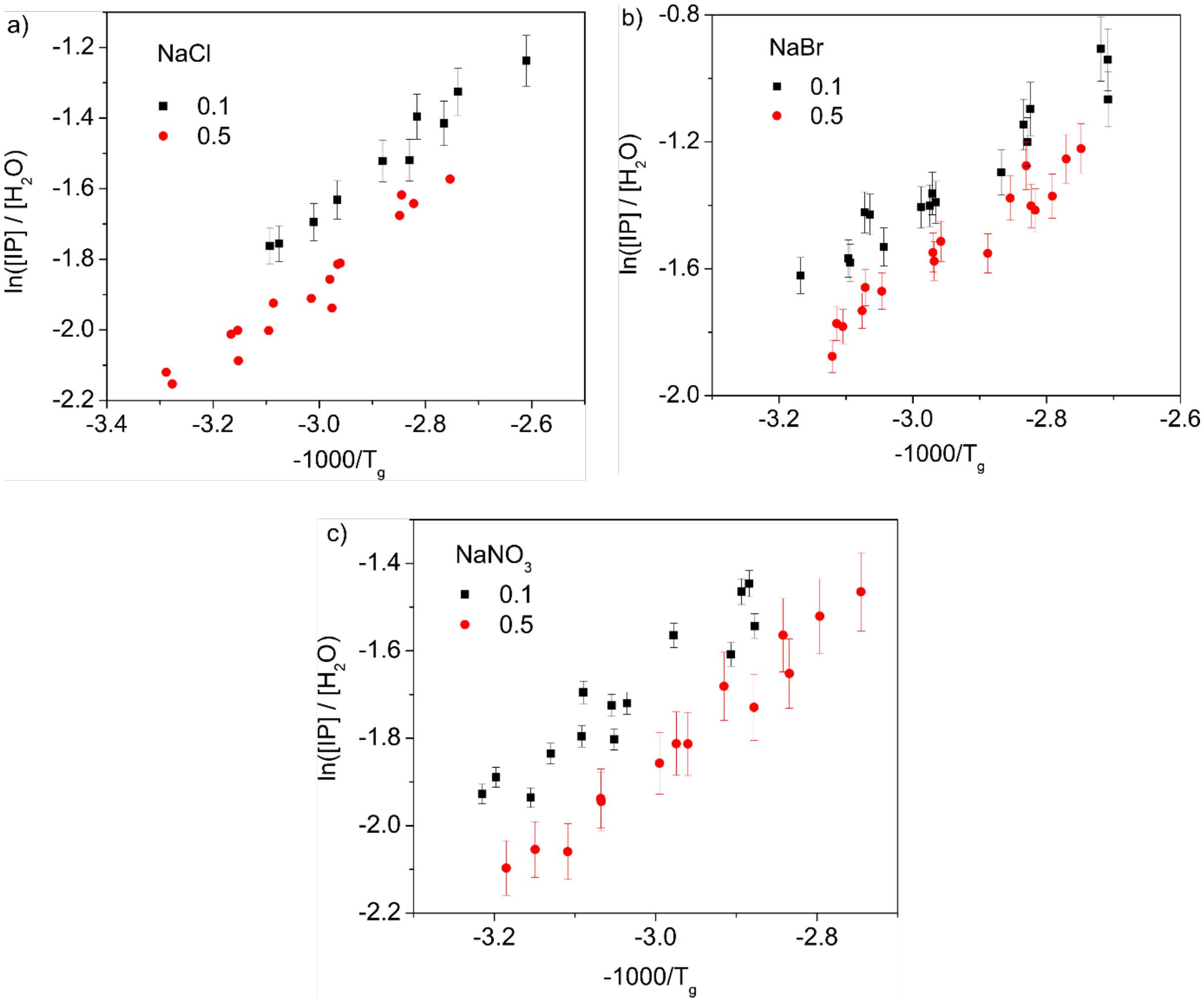

Figure S4. ln([IP] / [H_2O]) vs -1000/T_g for PECs prepared in a) NaCl b) NaBr c) $NaNO_3$. The R^2 value for linear fits for each are shown in **Table 2**.

Table S4. Critical fraction f calculated using Equation 10

Salt type/Salt concentration	y
NaCl / 0.1	0.11±0.02
NaCl / 0.5	0.17±0.03
NaBr / 0.1	0.07±0.02
NaBr/ 0.5	0.07±0.02
$NaNO_3$ / 0.1	0.09±0.03
$NaNO_3$ / 0.5	0.08±0.02
NaI / 0.1	0.10±0.02
